# Emerging Technologies in Algal Biotechnology: Toward the Establishment of a Sustainable, Algae-Based Bioeconomy

**DOI:** 10.3389/fpls.2020.00279

**Published:** 2020-03-17

**Authors:** Michele Fabris, Raffaela M. Abbriano, Mathieu Pernice, Donna L. Sutherland, Audrey S. Commault, Christopher C. Hall, Leen Labeeuw, Janice I. McCauley, Unnikrishnan Kuzhiuparambil, Parijat Ray, Tim Kahlke, Peter J. Ralph

**Affiliations:** ^1^Climate Change Cluster (C3), University of Technology Sydney, Ultimo, NSW, Australia; ^2^CSIRO Synthetic Biology Future Science Platform, Brisbane, QLD, Australia

**Keywords:** microalgae, synthetic biology, phenomics, industry 4.0, bioproducts, food, bioremediation, feedstock

## Abstract

Mankind has recognized the value of land plants as renewable sources of food, medicine, and materials for millennia. Throughout human history, agricultural methods were continuously modified and improved to meet the changing needs of civilization. Today, our rapidly growing population requires further innovation to address the practical limitations and serious environmental concerns associated with current industrial and agricultural practices. Microalgae are a diverse group of unicellular photosynthetic organisms that are emerging as next-generation resources with the potential to address urgent industrial and agricultural demands. The extensive biological diversity of algae can be leveraged to produce a wealth of valuable bioproducts, either naturally or via genetic manipulation. Microalgae additionally possess a set of intrinsic advantages, such as low production costs, no requirement for arable land, and the capacity to grow rapidly in both large-scale outdoor systems and scalable, fully contained photobioreactors. Here, we review technical advancements, novel fields of application, and products in the field of algal biotechnology to illustrate how algae could present high-tech, low-cost, and environmentally friendly solutions to many current and future needs of our society. We discuss how emerging technologies such as synthetic biology, high-throughput phenomics, and the application of internet of things (IoT) automation to algal manufacturing technology can advance the understanding of algal biology and, ultimately, drive the establishment of an algal-based bioeconomy.

## Introduction

By 2050, it is estimated that the world population will exceed 10 billion people (United Nations, 2019). Agriculture is already nearly maximally exploited, most arable land is already in use, and issues such as climate change and urban expansion pose important challenges to the future of agriculture (Foley et al., 2011). Simply increasing the intensity of agriculture, farming, fishing, and fossil oil extraction will not be sufficient to meet future demands. Rising global temperatures, extreme weather, changing climatic patterns, and loss of cultivable land will require drastic changes in current agrotechnology (Wurtzel et al., 2019) to minimize environmental impact through sustainable sourcing of commodities such as food, bioproducts, and bulk chemicals. Implementation of high-tech engineering and molecular genetics approaches, in the forms of phenomics and genetic engineering, has effectively improved the productivity, cost-effectiveness, and environmental impact of agricultural crops such as soy, corn, wheat, and rice (Mir et al., 2019). At the same time, plant-derived alternatives for animal-based foods such as meat and dairy, and commodities derived from petroleum such as plastics, are being developed (Zhu et al., 2016). Despite the clear advantages the of these solutions, the use of food crops to replace less sustainable manufacturing practices will eventually contribute to increased agricultural demand and face the same challenges that have been characterizing the “fuel vs. food debate.” Therefore, new solutions and additional resources are required to meet the increasing demands.

Photosynthetic microalgae are microbes that have colonized every habitat on Earth, and exhibit extraordinary biological diversity, estimated to be greater than 200,000 species (Guiry, 2012), which reflects an enormous range of ecological adaptations. Unlike other microbes often exploited for bio-based manufacturing, such as yeast and bacteria, phototrophic algae have the advantage to use sunlight to fix atmospheric carbon, reducing their reliance on sugars for fermentation. Naturally thriving in environments with intermittent and scarce nutrient availability, many species of microalgae have evolved efficient metabolic adaptations to grow rapidly under favorable conditions (Smetacek, 1999; Litchman, 2007). As a result, algae often have a higher photosynthetic efficiency than plants (Bhola et al., 2014), which translates into a higher capacity to generate biomass (Benedetti et al., 2018).

When grown at large scale – in either a pond or photobioreactor – microalgae are more water-efficient than crop plants (Demirbas, 2009) and can be cultivated on non-arable land with minimal use of freshwater (Demirbas, 2009), or even grow in seawater or wastewater. Thus, many geographical areas that are not suitable or sufficiently fertile for crop cultivation could be effectively used for large-scale algal cultivation. Many algal species are naturally efficient producers of carbohydrates, lipids, proteins, pigments, as well as a range of commercial secondary metabolites that are currently sourced from conventional agriculture (Koyande et al., 2019). Also, microalgae are emerging as a next-generation, cell-sized biofactories for the sustainable manufacturing of a myriad of products (Rasala and Mayfield, 2015; Vavitsas et al., 2018), following the example of established microbial platforms such as yeasts and bacteria. In this respect, microalgal biofactories have the potential to be less expensive and more sustainable platforms that may be naturally predisposed to produce certain plant-derived products (Vavitsas et al., 2018).

Currently algae are used for a relatively small number of industrial applications. Recent works have described in details the transition of the focus from algal-based bioenergy to high-value bioproducts, and the model of algae-based biorefineries (Laurens et al., 2017). In this review, we describe how recent landmark achievements have demonstrated the untapped commercial potential of algae-based applications. Specifically, we outline how cutting-edge technology developments such as automation, synthetic biology and phenomics can leverage the already naturally promising capabilities of microalgae in the coming years. By highlighting recent key achievements and unsolved knowledge gaps in the field – both in terms of technology advancements and applications – we describe the future development of microalgae as next-generation, low-cost, sustainable, scalable, and high productivity crop system. We anticipate that this will contribute to generate an algal-based bioeconomy, which will contribute to solutions to the imminent challenges caused by our growing society.

## Technology Development

**FIGURE 1:**
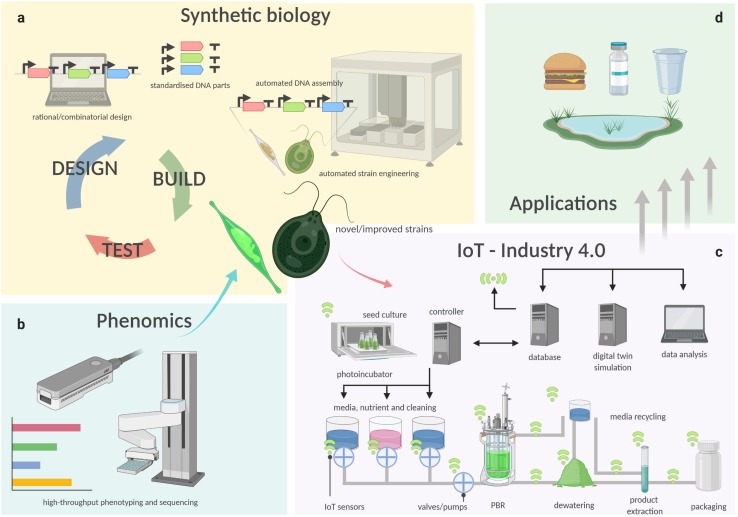
Schematic representation of how technologies such as synthetic biology **(a)**, phenomics **(b)**, cultivation technology and IoT **(c)**, are connected in a semi-automated pipeline for the manufacture of bioproducts from microalgae **(d)**. Biological functions are encoded into instructions through rational or combinatorial design of genetic constructs, which are then used to generate thousands of new microalgal genotypes with iteration of the *design-build-test* cycle **(a)**. Either natural isolates or engineered strains (*test* phase), are phenotyped in different, controlled conditions by high-throughput analyses. **(b)** Novel or improved strains with superior traits are then isolated and utilized for industrial production. **(c)** Using Industry 4.0 principles, in which a controller, such as an industrial programmable logic computer (PLC), receives information and logs its operation to a database computer. The database collects data from a network of plug-and-play sensors, which inform a digital twin simulation of the facility. The digital twin predicts the future demand and yield of the algae culture and updates the controller to optimize the process to match the predicted demand.

While crop plants have been bred and selected for millennia to isolate specific traits and to obtain highly productive strains, all present microalgal species are effectively environmental isolates. To maximize productivity and increase the industrial potential of microalgae, it is key to optimize both the organism and environment that supports its growth. In the following sections, we describe how this can be achieved through the latest technology developments in algal cultivation and harvesting, automation, phenotyping, and synthetic biology (Figure 1).

### Algal Cultivation

One of the most attractive intrinsic features of many algal species is that they are capable of rapidly and inexpensively generating large amounts of biomass compared to plants (Brennan and Owende, 2010). In nature, microalgae are capable of reaching high biomass concentrations under eutrophic conditions but, from a mass culture point of view, even these concentrations are not sufficient. In the past decade, there has been a large body of research focused on optimizing conditions that maximally promote algal growth rates, or elicit enhanced production of a specific product, under artificial growth conditions. However, one of the biggest limitations in algal mass cultivation is creating a cost-effective production system. In this regard, a diverse range of algal cultivation techniques can offer differing levels of control over the growth and product yield, with different associated capital and operating costs.

Several factors can limit microalgal growth in mass culture, including light availability, temperature and pH as well as both the concentration and ratio of the major nutrients, carbon, nitrogen and phosphorus (Sutherland et al., 2015). Some algae are capable of growing autotrophically as well as mixo- or heterotrophically which allows them to avoid light limitation constraints in dense culture, but does require the addition of organic carbon sources. As with any supplementation, adding organic carbon to the growth medium increases material input costs, but may achieve higher cell densities (Venkata Mohan et al., 2015). In principle, algae have the same basic requirements as plants, in that they need biologically available nitrogen and phosphorus, as well as trace nutrients (i.e. sulfur, calcium, iron, silicon.), and management of the pH levels to maximize nutrient availability (White and Ryan, 2015). The water source can affect what nutrients need to be additionally supplied, while water availability and recovery is key in determining what algal species can be selected. Algae can grow on different water sources, such as marine, fresh, or waste water. Wastewater is naturally rich in nutrients, but has additional contaminants that could cause culture crashes. There is a large diversity of marine algae and ready availability of seawater. However, seawater requires the addition of fertilizers and, in open systems, it is subjected to evaporation. This causes the salinity to be altered and to require monitoring. Fresh water may also require additional nutrients, but may also increase the strain on water supplies in water scarce regions. Recycling the water can aid in reducing these issues and improve the economic viability. The overall water requirement is still lower than traditional plant based crops (Rawat et al., 2013) leading algae to be desirable alternatives for cultivation.

Traditionally, microalgae have been grown in simple open ponds (Becker, 1994), but research and technological advances over the past several decades have led to a diversity of high-productivity bioreactor designs. Large-scale autotrophic algal production designs accommodate suspended or attached growth in either open or closed systems, or a hybrid of these, reviewed extensively elsewhere (Ugwu et al., 2008; Brennan and Owende, 2010; Harun et al., 2010; Christenson and Sims, 2011; Olivieri et al., 2014).

Besides stagnant ponds, the cheapest option for large-scale microalgal production is a shallow open pond raceway design that includes basic mixing. Compared to other photo-bioreactor (PBR) designs, they also have lower energy requirements, lower capital and operating costs, and can be built at a large scale (Brennan and Owende, 2010; Borowitzka and Vonshak, 2017). However, they generally have the lowest areal productivity (<10 g m^–2^ d^–1^ compared to >20 g m^–2^ d^–1^ in some PBRs) (De Vree et al., 2015). Some advances have been made improving productivity by modifying the design of open systems, such as high rate open ponds (HRAP), where increased baffles or more complex geometries improve the overall mixing pattern and ensure algae remain in the illuminated part of the water column (Christenson and Sims, 2011; Craggs et al., 2012). Companies worldwide are investing in this system, For example in Hawaii, this has generated over $US 10 million gross profits from biomass grown in open ponds (Maeda et al., 2018).

Another improvement in outdoor open cultivation are bioreactors for attached growth, such as algae turf scrubbers (ATS), or motorized wheels with biofilm growth (Wang and Lan, 2018). Novel biofilm-based algal cultivation in particular has seen increased research in recent years, in part due to higher harvested solid content (10–20% compared to <0.02% for suspended systems), which leads to lower harvesting costs. While biofilm growth is not suitable for all algal species, and can lead to complex mixed algae-bacteria communities (and therefore less suited for high-value, single products), it has been investigated for wastewater remediation (Gross et al., 2015) (section “Algal Biodegradation of Emerging Contaminants”). Closed suspended growth PBRs, such as flat-plate, tubular, or bag reactors have increased operating control, better mixing, and less chance of contamination compared to open systems and are suitable for genetically modified organisms, but also have significantly higher capital and operational costs (Gupta et al., 2015). Closed systems can be operated using artificial light (at increased costs), which can be tailored to the specific algae to increase productivity (Schulze et al., 2014; Glemser et al., 2016). In addition, genetically modified organisms (GMOs) may have regulatory limitations that prevent them from growing in open systems where they can be released into the wild. As such, closed systems may become increasingly common in the future. Despite these advantages, most of the current production is done in open pond systems (on the order of thousands of tonnes per annum) for products such as biofuels, animal feed, and nutraceuticals, while closed systems (hundreds of tonnes per annum) are used primarily for high-value products (Posten, 2009; Borowitzka and Vonshak, 2017).

Various techno-economic assessments have reviewed the feasibility of large-scale algal production (Laurens et al., 2017). The capital cost of an open pond can range from ∼$US 6/m^2^ (Craggs et al., 2012) to US$ 50/m^2^ (Huntley et al., 2015), while enclosed PBR systems can to cost up to 3 to 30 times more (Panis and Carreon, 2016). Operating cost can instead vary from US$ 0.8/kg dry weight (DW) to up to $8/kg DW for various systems and applications. Biofuels in particular have received most attention and are currently not price competitive yet, with production costs at ∼ US$ 3/L compared to <US$ 1/L of producing fuel from fossil oil (Sun et al., 2011; Laurens et al., 2017; Roles et al., 2020). The areal/volumetric productivity is generally one of the largest uncertainties as well as drivers for success (Jonker and Faaij, 2013; Chauton et al., 2015; Panis and Carreon, 2016; Hoffman et al., 2017). Strain selection is key to optimize productivities for the final product. This could require designing novel strains through genetic engineering or synthetic biology (section “Synthetic Biology”) that have been thoroughly profiled and selected using a phenomics approach (section “Phenomics”). On the cost side of the equation, dewatering is one of the major expenses in algal processing currently, consisting in up to 20–30% of the final cost, as reviewed extensively by Christenson and Sims (2011), Milledge and Heaven (2013), Gerardo et al. (2015) and Fasaei et al. (2018).

Algal suspensions are generally very dilute; therefore increasing the biomass content in the cultivation stage can substantially reduce costs, which is a major advantage for closed and attached growth PBRs compared to open ponds (Fasaei et al., 2018). Advances have been made in harvesting technology, by employing for example cross-flow filtration (Gerardo et al., 2014), cheaper flocculants (’T Lam et al., 2018; Nguyen et al., 2019), bio-flocculants (Ummalyma et al., 2017), microfluidics at lab scale (Kim et al., 2018), and novel techniques such as pulsed electric field, ultrasound, and electroflocculation, that have yet to be demonstrated at industrial scale (Milledge and Heaven, 2013; ’T Lam et al., 2018). However, while some harvesting processes can reduce the energy costs – for example filtration has a lower energy requirement compared to centrifugation – they can lead to a higher operating cost (e.g. filtration is subject to membrane fouling) (Bilad et al., 2014). Flocculants, chemicals added to cause algal cells to aggregate, can be inexpensive and have a long history in wastewater treatment, but can be hard to recover and affect downstream processing and media recycling (Milledge and Heaven, 2013). To address these short-falls, some systems combine multiple harvesting steps (e.g. flocculation combined with dissolved air flotation to remove the aggregates) (Pragya et al., 2013), while others are looking to bypass the harvesting step completely by modifying the algae to secrete the compound of interest (Christenson and Sims, 2011). The final harvesting step depends on the required product, the type of algae, and the specific cultivation strategies. As such, there is no one-size-fits-all solution to harvesting of the algal cultures.

In many cases, the final products can fit into existing industrial processes, for example transesterification for biofuels and extraction of high-value products (Greenwell et al., 2010; Khanra et al., 2018). There is also ongoing research for improvements in extraction of the algal products, such as by using supercritical extraction, pressure or microwave assisted extraction, ionic liquids, novel (less toxic) solvents, enzyme assisted extraction, or aqueous biphasic systems (Kadam et al., 2013; Kumar et al., 2015; Chew et al., 2017; ’T Lam et al., 2018; Khanra et al., 2018). Many of these novel and “green” extraction processes are dependent on the desired product, and are still only in use at the lab or pilot phase; getting them to an industrial scale would require significant investment and further research (Michalak and Chojnacka, 2015).

There is currently no single “best practice” method to cultivate algae, especially at scale. Final design of the system is dependent on the final product, the geographical location, as well as local resources available (e.g. accessibility to water, to CO_2_, and to waste streams). Modeling and lab scale experiments have suggested novel innovations in designing and operating the process, but the final consideration is the cost: some processes may have a larger up-front capital cost, but reduce the overall operating cost (e.g. HRAP ponds), while others may have very low capital costs but affect further downstream processing (e.g. chemical flocculation). As such, due consideration for the overall cost will guide the final design and operation of the system.

#### Industry 4.0 Approach to Algal Biorefineries

**FIGURE 2:**
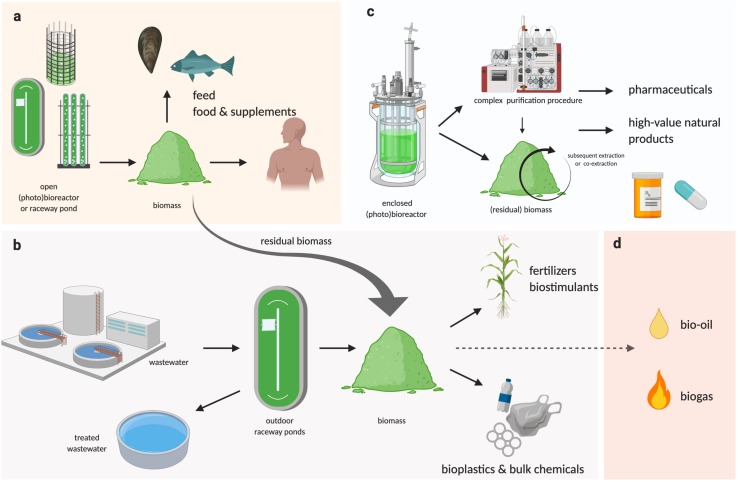
Schematic representation of a multi-product algal bio-refinery model. **(a)** Algal biomass cultivated at large scale in outdoor raceway ponds or large PBRs can be used as feed or food supplements, where the residual biomass and/or biomass generated from bioremediation processes can be used for industrial applications **(b)**, as well as for bioenergy (**d**, not reviewed here), while pharmaceuticals or other high-value products requiring complex and controlled extraction procedures could be co-extracted or subsequently extracted from algal biomass grown in enclosed bioreactors (BRs) or photobioreactors (PBRs) **(c)**.

Regardless how the biomass is produced, if the downstream processing can be performed in an integrated biorefinery that allows the greatest number of products and co-products to be extracted (Figure 2), and with the least amounts of residual/waste, it will ensure the maximum return on investment for downstream processing. Industry 4.0 is an advanced manufacturing approach based on machine-to-machine communication technologies, also known as “the Internet of Things,” or IoT (Atzori et al., 2010), whereby automation, sensors, and machine learning create a self-adapting manufacturing processes able to adjust in real time to changes in the process itself (Kagermann et al., 2011; Kagermann et al., 2013). In a microalgal biorefinery, this means that not only can the algal cultivation and harvesting system be automated to reduce operating costs, but a network of plug-and-play IoT sensors could allow the operators to monitor the algae growth and productivity in real time (Figure 1c; Whitmore et al., 2015). The concept of Industry 4.0 goes a step further by building a simulation, or digital twin, of the facility and the algal culture from the sensor data. The simulation can make real-time predictions of future cellular yield and adjust operations to meet expected product demand and to reduce waste (Figure 1c; Tuegel et al., 2011; Uhlemann et al., 2017; Tao et al., 2018). For example, a fully realized Industry 4.0 microalgal biorefinery (Figures 1c, 2) would link the controlled cellular yield of specific components with automated serial downstream extraction of several co-products that are driven by current demand, rather than traditional linear production stockpiling that awaits demand (Kagermann et al., 2013). Biorefineries can be located at regional hubs to service surrounding producers.

### Phenomics

Phenomics is defined as “the acquisition of high-dimensional phenotypic data in an organism-wide scale” (Houle et al., 2010). Algal phenomics is currently in very early stages of developments, however, it holds great potential in microalgal agriculture for food security (section “Food and Nutraceuticals”), bioproducts sourcing (sections “Food and nutraceuticals,” “Feedstocks,” “High-Value Products,” and “Biopolymers, Bioplastics, and Bulk Chemicals”), bioremediation (section “Algal Biodegradation of Emerging Contaminants”), and carbon sequestration. By creating a database of *GxE* = *P* [where *G*, genome, *E*, environment(s) and *P*, phenotype(s)] interactions for a given algal species, researchers can screen natural and artificial diversity for the combination of gene alleles that will combine essential phenotypes (Furbank and Tester, 2011), such as fast growth and high product yield.

Recent advancements from the field of plant phenomics highlight the potential impact of phenomics techniques and technologies in microalgae. For example, a recent phenomics-based study on *Arabidopsis thaliana* yielded a mutant exhibiting both increased pathogen defense and photosynthetic growth, breaking the supposed trade-off between growth and defense that was a near-dogma among plant researchers (Campos et al., 2016; Cruz et al., 2016). In microalgae, such a phenomics approach could create equally dramatic combinations of useful phenotypes, such as a strains that use quorum sensing (Das et al., 2019) to trigger autoflocculation (González-Fernández and Ballesteros, 2013) and induction of product synthesis (e.g. carotenoids) (Gong and Bassi, 2016) only when the culture reaches harvest density.

A major limitation to present-day microalgal phenomics is the lack of searchable phenomics databases. Plant and yeast researchers, for example, can design or even perform *in silico* experiments using The Arabidopsis Information Resource (TAIR) (Lamesch et al., 2011) and PROPHECY (Fernandez-Ricaud et al., 2005; Fernandez-Ricaud et al., 2016) databases, respectively. Such tools accelerate research by showing how different genes can be related by a shared phenotype (Ohyama et al., 2008), or even differentiate the functions of seemingly redundant gene copies (Yadav et al., 2007). To bring this power to the field of algal research, there will need to be investments in building a comprehensive phenotypic database. The field of plant phenomics has already created the standards for data sharing, knowledge retrieval, and ontology annotation (Oellrich et al., 2015; Munir and Sheraz Anjum, 2018; Neveu et al., 2019), which can be adapted to algae. The data analysis tools presently used for model microbes (e.g. yeast) can also be applied to microalgae to measure phenotype data from high-throughput algae culture formats (agar plates, microplates, etc.) using standard microbiology sensors such as fluorescence and absorption spectrophotometers (Fernandez-Ricaud et al., 2005; Fernandez-Ricaud et al., 2016), hyperspectral cameras (Roitsch et al., 2019), and flow cytometers (Cagnon et al., 2013). Even morphological phenotypes can be automatically digitized via machine learning (ML) approaches such as image processing with Support Vector Machines (SVNs) and Convoluted Neural Networks (CNNs), as demonstrated in Mohanty et al. (2016) and Sladojevic et al. (2016).

One challenge for developing microalgal phenomics databases will be choosing the growth environments that cause the microalgae to display a range of phenotypes based on their genetic predispositions. For example, much research has been done using high and low concentrations of carbon dioxide to learn about the roles of genes in photosynthesis (Suzuki et al., 1999; Vance and Spalding, 2005; Duanmu et al., 2009), but elucidating phenotypes related to stress responses and repair cycles can be more challenging (Cruz et al., 2016; Tietz et al., 2017). The risk of poorly chosen algal phenomics reference environments could result in insufficient segregation of phenotypes (Thomas, 1993), or worse, the environments might be so different from large scale cultivation as to render the measured phenotypes misleading and irrelevant to large scale enterprises (Rawat et al., 2013).

Another obstacle to algal phenomics is the limited capacity to manipulate the genetics of many non-model microalgal species. Many microalgae have complex life cycles (Graham et al., 2009) and their genomes are often large and highly repetitive, defying typical shot-gun sequencing techniques for genome sequencing and assembly (Paajanen et al., 2017). However, 3rd generation methods that allow long-read sequencing – such as Nanopore and PacBio sequencing – are breaking the log-jam. For example, PacBio sequencing has proven capable of mapping *trans-*gene integration sites in plants (Liu et al., 2019). These tools will be key in mapping genotypes of mutant libraries. Novel gene editing technologies such CRISPR-Cas9 are increasingly used to create large, genome-wide knock-out libraries in important crops such as tomatoes and rice (Jacobs et al., 2017; Meng et al., 2017). In the future, a combination of these gene-editing methods with 3rd generation sequencing technologies will enable cost and time-effective creation and mapping of knock-out libraries of important microalgal species. With a reference genome in hand, linking a phenotype to the relevant gene alleles traditionally relies on statistical analysis of the progeny after cross-breeding two individuals or populations with different phenotypes for example through Quantitative Trait Loci (QTL) mapping. With each successive generation, the genomic regions responsible for the phenotypes can be narrowed down until one has a testable list of candidate gene loci (van Bezouw et al., 2019). Given that many microalgae either do not breed at all or only under often unknown environmental conditions, novel approaches must be developed to fully utilize the power of phenomic mutant screens.

Once these challenges have been solved, algal phenomics will have a big impact on algal biotechnology by enabling the development of microalgae as new bioproducts and pharmaceutical workhorses (section “High-Value Products”). In this case, the yield of the desired product (or a suitable proxy) is treated as one phenotype in a phenomics search for both productivity and reliability among engineered strains. In addition, the maturation of algal phenomics tools will likely change the very mindset of algal biotechnology researchers. Presently, algal biotech researchers pick a single strain that synthetises the product of interest, and then they try to optimize the culture environment to improve the productivity, often resulting in expensive and complicated PBR design (Vasumathi et al., 2012; Wang et al., 2012; Melnicki et al., 2013; Lucker et al., 2014). In the era of algal phenomics, researchers will instead define their production culture environment, and optimize the algae to that environment, as much as a plant breeder would do for field crops (Donald, 1968; Jordan et al., 2011).

### Synthetic Biology

Synthetic biology applies engineering principles to the rational design of living organisms. Within this discipline, a biological system is viewed as a collection of characterized genetic parts that can be modified and reassembled to alter existing functions or to build them *de novo* in alternative host organisms. Genetic designs are revised through iterations of a design-build-test-learn cycle to achieve optimized metabolic configurations for biotechnological applications (Khalil and Collins, 2010; Nielsen and Keasling, 2016). Synthetic biology applied to microalgae will combine this powerful new approach with the benefits of a photosynthetic microbial host to generate novel production strains tailored to suit future environmental challenges (Figure 1a).

Tools for the genetic engineering of microalgae are evolving rapidly, enabled by the increased availability of sequenced genomes across multiple algal lineages. The sequencing of microalgal genomes has facilitated genetic tool development in the green alga *Chlamydomonas reinhardtii* (Mussgnug, 2015), stramenopiles *Phaeodactylum tricornutum* (Huang and Daboussi, 2017) and *Nannochloropsis gaditana* (Poliner et al., 2018), and cyanobacteria *Synechocystis* sp. PCC 6803 (Hagemann and Hess, 2018), among others. In addition, methods for the genetic transformation in microalgae have been optimized for many species and include natural transformation, electroporation, bead beating, biolistic transformation, and conjugative plasmid transfer (Qin et al., 2012).

Genomic data from these species have facilitated the identification of native genetic elements necessary for genetic engineering and successful transformation. Several constitutive and inducible endogenous promoter/terminator pairs have been demonstrated to effectively express transgenes in model species (Wang et al., 2012; Ramarajan et al., 2019), including bidirectional promoters for gene stacking or co-expression with a selectable marker (Poliner et al., 2018). In addition, heterologous or synthetic promoter sequences have been characterized (Berla et al., 2013; Zhou et al., 2014; Scranton et al., 2016). Other regulatory effectors, such as ligand-binding riboswitches, have been identified and developed as tools to regulate gene expression in cyanobacteria and *C. reinhardtii* (Moulin et al., 2013; Nakahira et al., 2013). Additional characterization of sequences that regulate transcription will be crucial to transition from stepwise genetic engineering to targeting multiple sites, introducing multi-gene pathways, or building independent synthetic circuits. In addition to sequences that modulate transcription, the molecular toolkit in microalgae also includes a useful suite of selectable markers, reporter genes, protein tags, and peptide sequences for ribosomal skipping or protein localization (Vavitsas et al., 2019). To standardize these commonly used genetic parts and to facilitate collaboration, the scientific community has adopted Type IIS restriction endonuclease cloning systems. This approach allows for efficient modular assembly of complex plasmids from a library of domesticated parts, and is being widely implemented in several models, including in plants (Patron et al., 2015). Suites of parts specific to microalgae have been developed to be compatible with a common syntax to benefit from existing part registries. Type IIS cloning systems specific to microalgae include the MoClo toolkit for *C. reinhardtii* (Crozet et al., 2018), CyanoGate for cyanobacteria (Vasudevan et al., 2019), and uLoop for diatoms (Pollak et al., 2019).

Multiple molecular techniques are available to modify native gene expression or target specific areas of the genome in microalgae. Gene knockdown by introduction of antisense, artificial small RNAs, and CRISPRi has been implemented in multiple systems (De Riso et al., 2009; Zhao et al., 2009; Yao et al., 2016; Wei et al., 2017; Sun et al., 2018). Site-specific genetic manipulation by homologous recombination (HR) is routine in cyanobacteria (Zang et al., 2007), the chloroplast genome of *C. reinhardtii* (Esland et al., 2018), and the nuclear genome of *Nannochloropsis* (Kilian et al., 2011). In contrast, HR occurs at a low frequency in the nuclear genome of *C. reinhardtii* and *P. tricornutum*, but can be induced in the presence of double-strand DNA breaks by targeted endonucleases, enabling targeted gene knockout and/or knock-in (Shin et al., 2016; Greiner et al., 2017; Kroth et al., 2018). Although technology for precision genome editing, including zinc-finger nucleases, transcription activator-like effector nucleases (TALENs), or CRISPR/Cas9, has been reported in many microalgae (Sizova et al., 2013; Weyman et al., 2015; Li et al., 2016; Nymark et al., 2016; Ajjawi et al., 2017), several challenges related to targeting, efficiency, and toxicity remain to be fully overcome. Strategies to circumvent these issues include transient Cas9 expression (Guzmán-Zapata et al., 2019), direct ribonucleoprotein (RNP) delivery (Baek et al., 2016; Shin et al., 2016) and use of Cas variants (Ungerer and Pakrasi, 2016). Marker-free and multiplex gene knock-out remains a challenge in some microalgae, although the use of multiple sgRNAs to multiplex genome editing targets has been shown to be feasible in diatoms (Serif et al., 2018).

Despite the rapid advances in the genetic tools available in microalgae, the field trails behind other established chassis microorganisms such as *Escherichia coli* and *Saccharomyces cerevisiae*. These model systems benefit from decades of intense study, resulting in diverse suites of characterized genetic parts and tools, known metabolic features, and well-annotated genomes. Approaches such as protein engineering and directed evolution that have been effectively implemented in these traditional hosts (Abatemarco et al., 2013) could also be applied in microalgae to hasten their development as chassis organisms.

Advances in synthetic biology are also enabling the design of entire microbial genomes (Hutchison et al., 2016; Richardson et al., 2017). While still on the horizon for eukaryotic algae, the development of self-replicating episomes in diatoms (Karas et al., 2015) has demonstrated that a synthetic sequence can be faithfully maintained in the diatom nucleus without integration into the native genome. This innovation is a step toward the design and assembly of independent, artificial chromosomes in microalgae. Reconstruction of a native *P. tricornutum* chromosome has already been demonstrated in yeast (Karas et al., 2013), and it is possible that a similar approach could be used to construct completely refactored chromosomal sequences.

Currently, genetic engineers are limited by the number of designs that they can feasibly assemble and test. However, it is anticipated that increased integration of computational design and automation with biology will rapidly shift this paradigm. Computational modeling can be used to predict non-intuitive approaches to optimize metabolic flux through heterologous pathways, as was demonstrated by the optimization of terpenoid production in cyanobacteria (Lin et al., 2017). Novel biological designs or complex combinatorial libraries can be rapidly assembled and evaluated in automated, high-throughput biofoundries, which are attracting investment from research institutions across the globe (Hillson et al., 2019). To evaluate clone libraries at scale, strain development must also be accompanied by improved technology for small molecule detection, such as the development of novel biosensors, as well as advancements in multi-dimensional phenotyping (section “Phenomics”).

Synthetic biology is not limited to the production of existing natural compounds, since the deconstruction of biology into its basic genetic components permits systems to be redesigned free from pre-existing constraints. An exciting avenue of synthetic biology will be the creation of novel, new-to-nature compounds with potential new functions and applications (Moses et al., 2014; Arendt et al., 2016; Luo et al., 2019). Synthetic biology can also be leveraged to improve agricultural outcomes for the cultivation of microalgae, including optimization of photosynthetic efficiency and improving carbon utilization (Gimpel et al., 2013; Erb and Zarzycki, 2016). For example, scenarios for the synthetic redesign of more efficient photosynthetic carbon fixation have been computationally predicted (Bar-Even et al., 2010). Given these advancements, the application of synthetic biology to microalgae has enormous potential to reinvent conventional animal and plant-based industries (e.g. food, high-value products, and chemicals) through innovations to minimize cost and environmental impact.

## Applications

### Food and Nutraceuticals

Increasing the current capacity of microalgae to provide a source of nutrients, minerals, trace elements and other bioactive compounds is an active area of research that establishes a precedent for the development for new health products (Plaza et al., 2008; Lordan et al., 2011; Wells et al., 2017; Barkia et al., 2019). The microalgal industry has yet to reach its full potential, with an estimated global net worth of $US1-1.5 billion (Pulz and Gross, 2004). Due to a history of safe production and consumption, the cyanobacteria *Spirulina* sp., along with the green algae *Chlorella* sp. and *C. reinhardtii* are internationally recognized as “generally regarded as safe” or GRAS, a certification legislated under the United States Food and Drug Administration (FDA, 2019). Other certified GRAS species include the green algae *Haematococcus* sp. and *Dunaliella* sp. (FDA, 2019).

There are many commercial food markets that can be occupied by ingredients and products derived from microalgal biomass. For example, microalgal biomass can be a source of bulk protein, carbohydrates, and lipids (Koyande et al., 2019). Microalgal protein is a particularly promising avenue to contribute to the future of sustainably based agriculture. Currently, the majority of global protein intake is attributed to higher plants (Billen et al., 2014; Henchion et al., 2017; Caporgno and Mathys, 2018), but plants require large amounts of arable land, water, and use of herbicides and fungicides (Dahman et al., 2019). Algal-sourced protein can be a sustainable alternative soy-based protein, due to its higher protein content and favorable amino acid profile, making it a high-quality protein for human nutrition (Spolaore et al., 2006; Kent et al., 2015). Recent studies show promising results with regard to improved physico-chemical and nutritional properties of *Spirulina* protein blends (Grahl et al., 2018; Palanisamy et al., 2019).

Some microalgae species are also a source of bioactive secondary metabolites that may ameliorate disease symptoms or causes, such as inflammation (Montero-Lobato et al., 2018) or provide protection to neuro-degenerative diseases (Olasehinde et al., 2017). Dried algal biomass from GRAS-certified species is most commonly consumed as a powder and already marketed as dietary supplement to improve health, is often added to other foods, such as blended beverages (Vigani et al., 2015). Powdered dietary supplements have been assessed in a number of clinical trials with some promising outcomes (Merchant, 2001; Karkos et al., 2011). For example, both *Spirulina* sp. and *Chlorella* sp. have clinically shown the ability to positively affect lipid profiles, various immune variables, and have antioxidant capacities (Mao et al., 2005; Park et al., 2008; Ryu et al., 2014; Kim et al., 2016; Garcia et al., 2017).

With an increased public preference for naturally sourced food additives, microalgae pigments offer an appealing alternative to synthetic pigments. Naturally derived pigments are a group of compounds that are inherently bioactive. They act as radical scavengers and can reduce oxidative damage (Singh et al., 2005), and therefore have appeal as dietary supplements or fortifying ingredients to promote human health (Stahl and Sies, 2005). This is in contrast to synthetic pigments, some of which are raising increasing concerns regarding their toxicities and subsequent adverse health effects, whilst also not providing any nutritional value (Oplatowska-Stachowiak and Elliott, 2017).

Microalgae are major producers of pigments such as fat-soluble chlorophylls, carotenoids (carotenes and xanthophylls) and water soluble phycobilins e.g. phycocyanin (Begum et al., 2016). *Haematococcus* sp. and *Dunaliella* sp. are two species that can accumulate significant levels bioactive pigment molecules such as astaxanthin (Guerin et al., 2003) and β-carotene (Tafreshi and Shariati, 2009), respectively. Astaxanthin is notable for its brilliant red color that brightens the flesh of seafood (Kidd, 2011). Humans do not synthesize astaxanthin, and dietary intake is almost exclusively via seafood (Kidd, 2011). Astaxanthin is presently mostly produced synthetically. Current production costs of microalgal-derived astaxanthin are still higher than those of synthetic (EUR 1540/kg and EUR 880/kg, respectively) (Panis and Carreon, 2016), although studies have estimated that these costs could be theoretically reduced to US$ 500 – US$ 800/kg (Li et al., 2011). Another common commercial pigment from microalgae is phycocyanin, derived from *Spirulina* sp. (Vigani et al., 2015). This deep natural blue pigment is utilized as a natural food colorant for food items such as chewing gum, ice sherbets, popsicles, candies, soft drinks, dairy products, and jellies (Begum et al., 2016).

Phytosterols are compounds often used as food supplement and cholesterol-lowering agents and are currently extracted from plants with suboptimal yields due to a complex extraction process (Ras et al., 2014). The sterol composition of algae is extremely diverse and comprises molecules typically synthetized by plants (e.g. brassicasterol and stigmasterol), animals (e.g. cholesterol) and fungi (e.g. ergosterol) (Rampen et al., 2010; Miller et al., 2012; Fabris et al., 2014; Lu et al., 2014), as well as novel and uncharacterized triterpenoids (Commault et al., 2019) and sterols, such as gymnodinosterol and brevesterol (Giner et al., 2003). Stigmasterol in particular is commonly used as cholesterol-lowering agents in food supplements (Batta et al., 2006). Several algal species naturally produce equal or greater amounts of phytosterols than plants, which are usually in the range of 0.025–0.4% of plant dry biomass (Piironen et al., 2003). Therefore diatoms and other algal groups have the potential to be alternative, low-cost, and more sustainable source of phytosterols (Ahmed et al., 2015; Jaramillo-Madrid et al., 2019). For example, the model diatom *P. tricornutum* produces up to 0.32% d.w. of phytosterols and the haptophyte *Pavlova lutheri* can accumulate phytosterols up to 5.1% d.w (Ahmed et al., 2015).

Essential polyunsaturated fatty acids (PUFA) such as eicosapentaenoic acid (EPA) and docosahexaenoic acid (DHA), play crucial roles in human health. DHA is necessary for neural development (Swanson et al., 2012) and is routinely utilized in infant formulas, fortified food and beverages and dietary supplements (Ratledge, 2004). Presently, most DHA and EPA is supplied by wild-catch and captive based fisheries (Lenihan-Geels et al., 2013). As primary producers of essential PUFAs in nature, with DHA concentrations up to 50% of total biomass (Ratledge, 2004), microalgae represent a promising and more sustainable alternative (Ryckebosch et al., 2014). Presently, the production costs associated with microalgal derived EPA/DHA reach US$ 40/kg EPA + DHA, but technological advancements could possibly lower this to ∼US$ 10/kg EPA + DHA, which is competitive if compared to fish oil (∼US$ 8/kg EPA + DHA) (Chauton et al., 2015).

Currently, three different commercial fermentation processes are used to produce DHA, with each utilizing different microorganisms (Ratledge, 2004). Martek Biosciences Corp (Netherlands) led the infant formula DHA market until 2011, when it was acquired by Dutch State Mines (DSM) in 2011. DSM utilizes the dinoflagellate microalgae *Crypthecodinium cohnii*, which accumulates DHA up to 60% of the total fatty acids fraction (Jacobsen et al., 2013) for use in infant formula, and *Schizochytrium* sp., a heterotrophic protist which can yield about 40% (w/w) of DHA (Ratledge et al., 2010). Oil from *Schizochytrium* sp. has been traditionally used for improving animal feeds, but there is also a market push toward human nutritional supplements (Ratledge and Cohen, 2008). The thraustochytrid *Ulkenia* sp. utilized by Nutrinova GmbH (Germany) produces up to 46% (w/w) DHA. In contrast to phototrophic algae, thraustochytrids are grown heterotrophically in stainless steel fermenters using complex organic substances, including by-products from other processes (e.g. sugars, organic acids) as a sole carbon and energy source (Chang et al., 2014). The oils obtained from *Schizochytrium* and *Ulkenia* are defined as “novel foods” under the European Union (EFSA Panel on Dietetic Products, Nutrition and Allergies [NDA], 2014) and in 2017 the Food Standards Australia New Zealand (FSANZ, 2019) approved the use of *Schizochytrium*-derived DHA-rich oil for use in infant formula products (Schedule 25, Permitted novel foods). Since 2017, several GRAS notices have been approved for *Schizochytrium-*derived oils by the Food Safety Authority (FDA), indicating a growing momentum in the utilization of microbe derived oils.

In contrast to DHA, much less progress has been made in the utilization of phototrophic microalgae for developing an alternative to fish oil for EPA and other important fatty acids. High-quality EPA is found in marine microalgae across a number of classes including Bacillariophyceae (diatoms), Chlorophyceae, Chrysophyceae, Cryptophyceae, Eustigamatophyceae and Prasinophyceae (Wen and Chen, 2003). While EPA production to date has focused on photoautotrophic growth, it is not yet economical, but emerging production and processing technologies may lead to sufficient enhancement of EPA production in microalgae to achieve market viability (Vazhappilly and Chen, 1998).

Whilst the microalgal industry does currently contribute biomass for food and nutrition, its scope is limited to a handful of algae and applications. Progress toward a greater utilization of algal products faces numerous technological challenges. Extensive screening and biological evaluation is needed to optimize production of specific metabolites and gain an understanding of how algal dietary value is affected by geographical region and growth season (Wells et al., 2017). Emerging technologies such as phenomics (section “Phenomics”) are useful to survey multiple quantitative traits and to provide feedback on culture optimization. Furthermore, progress in cultivation technology (section “Algal Cultivation”) and bioprocessing is needed to ensure such processes are economical viable and can compete with traditional and synthetic sources.

Further challenges include compliance with legislation, cost of production, and consumer perception. The latter will need to necessarily address issues regarding the association between microalgae and toxic cyanobacterial blooms, and their portrayal by the media. New algal strains without a documented history of safe consumption must be assessed and approved as “novel food” under EU and AUS legislative regulations (Sidari and Tofalo, 2019) or obtain a Food and Drug Administration (FDA) GRAS certification (Caporgno and Mathys, 2018), to be considered as future agricultural sources for food and nutrition. Factors such as health and nutritional benefits, taste, safety, freshness, and sustainability may persuade adoption of such products. Barriers such as lack of knowledge and familiarity must also be recognized before achieving consumer confidence. Future research will need to validate health benefits scientifically via robust clinical *in vivo* studies (i.e. random controlled trials), as well as directing efforts toward positive re-enforcement between new microalgal derived products and existing bio-products to help overcome negative consumer perceptions.

Thus, with efforts being made in emerging technologies such as phenomics and bioprocessing, microalgae is anticipated as promising future agricultural crop to cater for the increasing demands of future human and animal nutrition or other high value ingredients.

### Feedstocks

While the potential of algae as next-generation of biological resources is still emerging, some well-established industrial sectors are already routinely using them as feedstock. Among these, the aquaculture industry has used algae for the production of “aquafeed” for decades (Hemaiswarya et al., 2011). The rapid growth rates and balanced nutritional value of microalgae are ideal for aquafeed, and aquaculture production facilities commonly utilize microalgae either directly as live feed or indirectly as algal meal, consisting in the residual biomass left after extraction of lipids (Borowitzka, 1999).

The recent demand for algal meal is mainly driven by the increasing consumer demand in more sustainable food products. Currently, a major proportion of conventional agriculture and aquaculture utilizes fishmeal, the crude flour obtained after cooking, drying, and grinding fish parts, which has a high protein and PUFA content, and relatively low cost production (approx. $US1,500 per ton, source^1^). Fishmeal has been used historically as a feed for farmed seafood, poultry, and pigs, and even as a fertilizer (Hardy and Tacon, 2002). However, fishmeal is now widely recognized as unsustainable, as its production is largely based on by-catch, leading to depletion of ecosystems and the collapse of local fisheries. Therefore, more sustainable ingredients are increasingly considered as alternatives to fishmeal, including soybean meal (Alvarez et al., 2007), cottonseed meal, insects meal, legumes, and algae (Hardy and Tacon, 2002). While algal feed represents one of the most promising alternative to fishmeal, because of the low land, freshwater and carbon footprints (Kim et al., 2019), matching its production with the low cost and large scale of conventional fishmeal production (6 to 7 million metric tons per annum) has proven challenging. In this respect, future developments in large-scale culture systems, as detailed in section “Algal Cultivation,” but also in new business models incorporating multiple products (Figure 2), could help to solve these challenges and to achieve the potential of algal meal as an emerging feedstock. For example, large feeding trials have showed that, even after extraction of a vast majority of its PUFA content (which can be sold separately as high-value nutraceutical, section “Food and Nutraceuticals”), the residual biomass of *Nannochloropsis* has great potential as aquafeed for Atlantic salmon, common carp and whiteleg shrimp (Kiron et al., 2012). This clearly demonstrates the potential of algal meal, especially when integrated in a multi-product biorefinery business model, as an emerging and viable feedstock (Figure 2).

The most historical and natural application of algae for production of feedstock is as direct live feed. Indeed, algae have been widely used as direct live feed during juvenile stages of abalone, crustaceans, fish species and bivalves for decades (Benemann, 1992). Among these, bivalve hatcheries require the most microalgal production in comparison to any other form of food in aquaculture, due to bivalves being obligate filter-feeders throughout their entire life (Guedes and Malcata, 2012). Consequently, mass production of microalgae can account for >30% of a bivalve hatchery operating costs, indicating it is a major financial consideration for this sector of aquaculture industry (Guedes and Malcata, 2012). Approximately 20 algal species have been identified in the 1980s as most suitable live feed for aquaculture industry (Laing, 1987). From these selected species, the genera *Chaetoceros, Tisochrysis, Pavlova* and *Tetraselmis*, are considered some of the most suitable for the rearing of bivalves, with their specific size being one of the most important attribute (Guedes and Malcata, 2012). For example, *Tisochrysis lutea* (3–7.5 μm) is utilized throughout the production of many bivalves, from larvae and juveniles through to adults, as they are primarily an appropriate size but are also nutritionally valuable and robust in culture (Bendif et al., 2013). While *T. lutea* is suitable for all growth phases of bivalves, some other species such as the diatom *Chaetoceros muelleri* are slightly larger (5–8 μm) and therefore unsuitable for the early juvenile phase (Pacheco-Vega and Sánchez-Saavedra, 2009). As a result, the efficiency of feed profiles for bivalves has largely been optimized for the aquaculture industry by mixing a range of microalgae species that are nutritionally diverse and covering a range of sizes (Heasman et al., 2000). The biological differences between microalgae species means that their photosynthetic demands (light and CO_2_) and nutritional requirements are likely to differ significantly (Ihnken et al., 2011). However, a similar set of standard growth conditions are generally set for all cultures (Heasman et al., 2000). This “standardization” operated by the aquaculture industry implies that a largely un-optimized “one size fits all” culture system is employed, regardless of efficiency (Guedes and Malcata, 2012). Consequently, future biotechnological research (sections “Algal Cultivation,” “Phenomics,” and “Synthetic Biology”) integrating (i) differences in optimal growth conditions between microalgae species, (ii) strain selection, and (iii) new cultivation technology, especially the next generation of nearly fully automated photobioreactors, are likely to increase microalgal yields to an extent that was previously unimaginable.

### High-Value Products

Most of the high-value products that are currently sourced from higher plants are also naturally produced by algae, or could be produced by algae through genetic engineering and synthetic biology. Given the vast diversity of microalgae, they naturally produce an extremely wide – and largely uncharacterized – range of natural products, potentially useful for human consumption and use. Some products are already synthetized efficiently, while the yields of others can be maximized to meet industrial requirements by the integration of advanced strain and bioprocess engineering (sections “Algal Cultivation,” “Phenomics,” and “Synthetic Biology”). Only a minute fraction of all algal species, consisting of mostly model species, are currently profiled for their biochemical capabilities (Sasso et al., 2012). Therefore, the full potential of algae in this context can only be estimated. Besides the above-mentioned food supplements, pigments and PUFAs (section “Food and Nutraceuticals”), a substantial number of different high-value products are already being sourced from algae.

Plant biostimulants (PBs) are a heterogeneous class of compounds that include phytohormones, small molecules, and polymers, which are used to improve crop performances and protect from abiotic stresses (Drobek et al., 2019). Extracts and bioactive compounds derived from wild type microalgae species are increasingly being explored as source of PBs (Chiaiese et al., 2018). Although this application is still at early stages, it could be developed and result particularly advantageous if included in a multi-purpose algal biorefinery (Figure 2). As the biochemical composition of microalgae greatly varies depending on species and culture conditions, the potential of algae-based applications in this context could be leveraged by more detailed knowledge on algal biochemistry and physiology, to allow better choice of species and growth conditions to promote the production of specific PBs (Drobek et al., 2019). On the other hand, the potential of engineered algal strains to enable the production of higher amounts or specific molecules is completely unexplored. Advanced genetics and synthetic biology techniques could soon enable the development of novel algal strains designed to produce specific metabolites, phytohormones, or peptides with PBs activity.

Microalgae can be genetically engineered to synthesize a myriad of high-value products. Terpenoids are the largest class of natural products that include countless bioactive plant secondary metabolites with applications as cosmetics, biofuels, nutraceuticals, and as life-saving pharmaceuticals in high demand (Vickers et al., 2017). As plant secondary metabolites, these compounds are typically produced in trace amounts; therefore industrial extraction *ex planta* requires very large quantities of biomass, with high economic and environmental costs. This could be averted with microalgae engineered to produce these chemicals in higher concentrations than is possible in plants (Arendt et al., 2016; Moses et al., 2017; Vavitsas et al., 2018; Lauersen, 2019). Cyanobacteria, for example, have been widely used for heterologous plant-derived terpenoid engineering, extensively reviewed in Chaves and Melis (2018) and Lin and Pakrasi (2019), and proof-of-concept works have unveiled the potential of engineered eukaryotic microalgae such as *C. reinhardtii* in producing high value terpenoids such as the food flavoring and aromas sesquiterpenoid patchulol and (E)-α-bisabolene (Lauersen et al., 2016; Wichmann et al., 2018) and diterpenoids such as casbene, taxadiene, and 13R(+)manoylnyl oxide (Lauersen et al., 2018), as well as lambdane diterpenoids (Papaefthimiou et al., 2019), which are relevant precursors of plant-derived therapeutic and cosmetic products. Similarly, the diatom *P. tricornutum* is currently being explored for similar applications and demonstrated its potential in producing triterpenoid lupeol and traces of betulin, precursors of the topoisomerase inhibitor betulinic acid (D’Adamo et al., 2019), commonly used in anticancer and antiviral pharmaceutical preparations and naturally produced in trace amounts from the bark of plant species, such as the white birch tree (Pisha et al., 1995).

Plant monoterpenoids are particularly challenging compounds to produce in conventional microbial hosts such as *S. cerevisiae* and *E. coli* because these organisms do not naturally accumulate pools of the precursor geranyl diphosphate (GPP) (Vickers et al., 2017). It has been recently demonstrated that *P. tricornutum* naturally accumulates cytosolic pools of GPP and that these can be efficiently converted into the monoterpenoid geraniol (0.309 mg/L), through the episomal expression of a heterologous plant geraniol synthase enzyme (Fabris et al., 2020). Geraniol has several commercial applications as component of essential oils, flavouring agent and insect repellent, and is the key precursor of the monoterpenoid indole alkaloids (MIAs), a diverse group of bioactive plant metabolites that include the anticancer agents vinblastine and vincristine (Miettinen et al., 2014). This is a relevant demonstration that diatoms might harbour an important intrinsic advantage over conventional terpenoid production hosts for the synthesis of this challenging class of compounds, and that extrachromosomal episomes are suitable for metabolic engineering applications in diatoms, which is seminal for more complex, engineering approaches.

Exciting progress in algal genetics and synthetic biology, including key technologies for the assembly and expression of multi-gene constructs and tools for targeted gene editing (section “Synthetic Biology”), as well as advances in metabolic systems biology, will rapidly enable the expression of more complex metabolic pathways (Slattery et al., 2018) and increase understanding of the resulting interactions with endogenous algal metabolism, resulting in tailored engineering efforts that will go beyond simple proofs of concept and result in industrially relevant product yields.

Other products that could be sourced from engineered algal strains include industrial recombinant enzymes (Rasala et al., 2012; Lauersen et al., 2013) and protein-based therapeutics (Rasala et al., 2010; Gimpel et al., 2015). These recombinant protein drugs are generally produced in microbes such as *E. coli*, yeasts or mammalian cell lines such as Chinese Hamster Ovarian (CHO) cells. The latter, in particular, are associated with extremely high cost of production, mostly due to complex growth media composition (US$10 – 500/L) (Xu et al., 2017). Therefore, in the last two decades, an increasing research effort has been put into developing robust, alternative production hosts. Plant and algae-based expression systems are envisioned as a valid, low-cost solution for producing therapeutics in countries and areas that lack resources for costly mammalian-based fermentation systems (Taunt et al., 2018), with the advantage of being immune to most pathogens and contaminations that affect animal hosts (Specht and Mayfield, 2014). In this search, it has been demonstrated the suitability of *C. reinhardtii* to produce – predominantly in the chloroplast – functional recombinant therapeutics, including a fully assembled human antibody, immunoglobulin G (IgG) (Tran et al., 2009), vaccine subunits (Gregory et al., 2013), vaccine antigens (Demurtas et al., 2013), immuno-conjugated cytotoxins for cancer targeted treatments (Tran et al., 2013), and single domain antibodies (VHH) (Barrera et al., 2015). Diatoms such as *P. tricornutum* have also been used to successfully and efficiently produce and secrete fully assembled antibodies (Hempel et al., 2011b; Hempel et al., 2017), while the silicified *Thalassiosira pseudonana* has been engineered for targeted drug delivery *in vivo*, by displaying a recombinant IgG binding domain on the silica frustules, turning the whole cell into a drug delivery vector effective on tumor models (Delalat et al., 2015). The production of edible vaccines is another developing field where algae-based expression systems are finding a relevant niche (Specht and Mayfield, 2014), with particular relevance to the poultry and aquaculture industry. However, challenges will need to be addressed to make algae the preferred production hosts for therapeutics. In addition to the cultivation challenges already mentioned, the production of recombinant therapeutics in algae is currently hindered by overall low expression levels, and it is expected that developments in algal genetics and synthetic biology (section “Synthetic Biology”) will enable more competitive yields. Strategies involving innovative genetic design – for example the insertion of intronic sequences in the transgene of interest – could be used to significantly improve the expression of recombinant proteins (Baier et al., 2018). Also, to be suitable for therapeutics production, algal production hosts need to exhibit the correct post-translational modifications, such as protein glycosylation, to avoid adverse immune reactions in inoculated animals. Little is known about the *N-*glycosylation properties of microalgae, but large-scale profiling of glycosylation properties of diverse non-model species and genetic engineering may possibly offer possibilities for algae to become a preferred production platform for glycoproteins (Mathieu-Rivet et al., 2014).

From these convincing proof-of-concepts examples, the (enhanced) production of endogenous and heterologous high-value products in microalgae will enormously benefit from the technological developments reviewed in section “Technology Development.” More complex synthetic biology approaches, in combination with detailed knowledge on novel or engineered strains from high-throughput phenomics approaches, and advancements in cultivation technology (Figure 1), will address the main bottlenecks of low yields and upscaling, and open the doors to the cost-effective production of a much wider diversity of bioproducts. This will alleviate the environmental impact imposed by current practices involving inefficient bioproduct sourcing from plants or from other high-cost and less environmentally friendly production methods.

### Biopolymers, Bioplastics, and Bulk Chemicals

The demand of plastic and plastic-based products have grown significantly in last few decades, which has placed a major strain on the remaining petrochemical resources of our planet. The increasing production of these petrochemical-based plastics has also generated concern regarding plastic pollution worldwide, mostly in marine ecosystems due to their persistence in environment as non-biodegradable materials (Tetu et al., 2019). Therefore, alternatives to petrochemical-based plastics sources are in high demand, as they would make plastic production sustainable while mitigating the issue of plastic pollution. Algae have the potential to be an economically viable feedstock for bioplastics production, as the biomass can be sold at US$ 970/tonne, which is within the current standard range for other sources of bioplastics (US$ 800 – 1200/tonne (Beckstrom et al., 2020).

Microalgal biomass components such as starch, carbohydrates, and lipids can be converted into plastics (Noreen et al., 2016). There are currently three main approaches to produce bioplastics from microalgae, including: (i) direct use of microalgae as bioplastics, (ii) blending of microalgae with existing petroleum-based plastics or bioplastics, and (iii) genetic engineering of microalgae to produce bioplastic polymer precursors. In the first approach, Zeller et al. (2013) have reported production of bioplastics and thermoplastic blends directly from *S. platensis* and *C. vulgaris*, while Wang et al. (2016) described the preparation of thermoplastics by blending a heterogeneous population of planktonic algae. However, the most common approach to making microalgae-based bioplastics is to blend the biomass with existing petrochemical-based plastics, such as polyethylene, polypropylene, polyvinyl chloride. Shi et al. (2012) described the processing of microalgae-corn starch-based thermoplastics using *Nannochloropsis* and *Spirulina*, and further blending with polyethylene and polypropylene. *Chlorella* sp. biomass was blended with polyethylene and polypropylene and was found to possess good thermoplastics processability because of the presence of natural cellulosic type materials (Zhang et al., 2000a). The properties and processing of PVC-*Chlorella* composite has also been reported (Zhang et al., 2000b).

With the increasing demand of bioplastics in the market, considerable research effort has been directed in investigating the blending of algal biomass with other bio-derived plastics components. A recent study has reported of addition of green, brown, and red algal biomass to polylactic acid plastics (Bulota and Budtova, 2015) with no pre-treatment other than drying and sieving. Polyhydroxyalkanoates (PHAs), one of the widely studied biodegradable polyesters with high mechanical strength and melting point, is naturally produced in certain bacteria, including some cyanobacteria (Sudesh et al., 2000). Microbial production of PHAs generally occurs under stressful environmental conditions (Bassas et al., 2008; Balaji et al., 2013). PHA is generally extracted by three subsequent steps of disrupting the cells (by chemical, physical or biological treatment), recovery of PHAs, and purification (Fiorese et al., 2009). However, with the continuous increase in interest in PHAs production, metabolic engineering, and synthetic biology (section “Synthetic Biology”) could enable the heterologous synthesis of PHA precursors in eukaryotic microalgae as demonstrated in diatoms (Hempel et al., 2011a).

Biomass-derived chemicals, such as 5-hydroxymethylfurfural (5-HMF), levulinic acid, furfurals, sugar alcohols, lactic acid, succinic acid, and phenols, are considered platform chemicals. These platform chemicals are used for the production of a variety of important chemicals on an industrial scale (Kohli et al., 2019). Bio-based bulk chemicals possess a clear substitution potential for fossil oil-based bulk chemicals. However, current biomass feedstocks for industrial use are typically derived from plant material, posing challenges such as destruction of rainforests, competitive food consumption, and other adverse environmental impacts. Microalgae, with its superior areal productivity to traditional agricultural crops and high concentration of lipid, carbohydrate, and proteins, have appeared as an alternative and attractive candidate for the production of bulk chemicals, including bio-based platform chemicals and bio-based solvents (Wijffels et al., 2010). Catalytic valorization is an emerging field that can be applied to the production of value added chemicals from microalgae. Even though the technology readiness for commercialization is still a challenge, the field is active with several research groups working on algae and catalytic systems for the conversion of algal biomass to value added platform chemicals. For example, *Chlorococcum* sp. was reported to be converted into 1,2-propanediol (1,2-PDO) and ethylene glycol (EG) in water over nickel-based catalysts (Miao et al., 2015), while the hydrolysis of *Scenedesmus* sp. over the Sn-Beta catalyst was used to produce lactate (Zan et al., 2018). This was achieved via formic acid induced controlled release hydrolysis, with an achieved yield of 83%. Another recent study demonstrated the conversion of algal polysaccahrides from *Phorphyridium cruentum* and *C. vulgaris* to monosaccharides, HMF, and furfural in the neat deep eutectic solvent (DES) or in the biphasic system ChCl/oxalic acid/methyl isobutyl ketone (Bodachivskyi et al., 2019).

Microalgal biomass that has the lipids already extracted is good source for carbohydrates. The reported yields are up to 80% of the cell mass and hence, could be useful upon hydrolysis to generate fermentable sugars. A recent study has reported hydrolyses of lipid extracted *C. vulgaris* biomass using solid acid catalysts to obtain monosaccharides such as glucose, galactose, xylose, rhamnose, mannose, and 2,3 butanediol (Seon et al., 2019). These monosaccharides can be used for microbial fermentation to produce many useful products, such as lactic acid, hydrogen gas, and ethanol. 2,3 butanediol is a value-added chemical with great potential for the industrial production of synthetic rubber, plastic, and biosolvent (Soo-Jung et al., 2017; Seon et al., 2019). In another study, microalgal hydrolysate from *C. vulgaris was* converted into ethanol via continuous immobilized yeast fermentation at a yield of 89% (Kim et al., 2014).

Several challenges will need to be addressed in terms of low product yield and relatively high costs of such biochemical conversion processes. However, the integration of these application in a multi-product biorefinery approach (Figure 2), could improve the overall economic feasibility of bioplastic and bulk chemical production from microalgal biomass.

### Algal Biodegradation of Emerging Contaminants

Emerging contaminants (EC) are primarily synthetic organic chemicals, such as pharmaceuticals, herbicides, pesticides, and flame retardants, whose presence in the environments are of concern due to their potential risks to ecosystems and human health, at environmentally relevant concentrations (Petrie et al., 2015; Tran et al., 2018; Sutherland and Ralph, 2019). There is increasing concern over the presence of ECs in agricultural land- and water-scapes. With climate change and expanding populations, accumulating ECs due to agricultural intensification and increased water reuse could lead to unpredictable long-term consequences for humans and the environment (Martinez-Piernas et al., 2018). While direct application can be managed through improved on-farm best management practices, indirect application is reliant on improvements in wastewater treatment that would reduce, transform, or eliminate ECs.

Wastewater treatment using microalgae for nutrient removal is a well-established technology that has lower capital and operational costs, and is more efficient than traditional wastewater treatment systems (Benemann, 2008; Craggs et al., 2012). However, there have been few studies to date on the use of microalgae for bioremediation of ECs despite their potential for detoxifying organic and inorganic pollutants. Coupling of nutrient and EC removal by microalgae has the potential to provide more cost-effective and efficient wastewater treatment as well as meeting both environmental and human health protection goals (Sutherland and Ralph, 2019).

While still in its infancy, microalgal biodegradation provides one of the most promising technologies to transform, neutralize, or eliminate ECs from agricultural runoff. Unlike other remediation techniques, such as activated carbon adsorption filters, which simply concentrates the EC and removes it from one environment to another environment, biodegradation involves the transformation of complex compounds into simpler breakdown molecules through catalytic metabolic degradation (Sutherland and Ralph, 2019). Microalgal degradation of ECs can occur via two main mechanisms. The first mechanism involves direct metabolic degradation of the EC by the microalga. In this case, the microalga employs mixotrophic growth strategies and the EC serves as the carbon source or electron donor/acceptor (Tiwari et al., 2017). The second mechanism involves indirect, or co-metabolism, where the EC is degraded by enzymes that are catalyzing other substrates present (Tiwari et al., 2017). Microalgae possess a large number of enzymes that play a role in cellular protection through the deactivation and/or degradation of a range of organic compounds that induce cellular stress in microalgae (Wang et al., 2019). Microalgal degradation of ECs relies on a complex enzymatic process involving a number of enzymes, including: superoxide dismutase, catalase, glutamyl-tRNA reductase, malate/pyruvate dehydrogenase, mono(di)oxygenase, pyrophosphatase, carboxylase/decarboxylase, dehydratase, alkaline and acid phosphatase, transferase, and hydrolases (Elbaz et al., 2010; Xiong et al., 2018; Wang et al., 2019). Several of these enzymes, including superoxide dismutase and catalase, have shown increased activity in several freshwater microalgal species, when the cells were exposed to the veterinary antibiotics Florfenicol and Ofloxacin (Wang et al., 2019).

In one bioremediation study, the green algae *Scenedesmus obliquus* and *Chlorella pyrenoidosa* were found to enzymatically degrade progesterone and norgestrel by reduction (hydrogenation), hydroxylation, oxidation (dehydrogenation) and side-chain breakdown (Peng et al., 2014). In another study, co-metabolic removal of the antibiotic ciprofloxacin by the green alga *Chlamydomonas mexicana* was observed, but the enzymatic mechanisms involved in its metabolism were not identified (Xiong et al., 2017). Due to the complexity of enzymatic biodegradation processes, simply screening microalgal strains for EC biodegradation activity remains the most viable strategy for developing new bioremediation strains (Sutherland and Ralph, 2019).

One of the challenges with screening microalgae for EC biodegradation is the large number of both ECs and microalgal species. Currently, there are approximately 200 known ECs in the environment, while there are thousands of recognized algal species (Pradhan and Rai, 2001; Guiry, 2012). Therefore, there is a need for the development of cost effective high through-put screening methods that allow for rapid screening of a wide range of microalgal species against a wide range of ECs. A microalgal phenomics facility (section “Phenomics,” Figure 1b) would provide the necessary cost-effective and efficient high through-put screening to help rapidly develop microalgal biodegradation technology.

Another challenge with screening microalgae for EC biodegradation is that the enzymes responsible for degrading the EC may not be active at the time of screening (Sutherland and Ralph, 2019). This is due to both the production and maintenance of these complex enzymes being metabolically expensive, which comes at the cost of growth and reproduction of the cell (Sutherland and Ralph, 2019). For example, both the cellular energy budget and growth rates were significantly reduced in the microalga *Raphidocelis subcapitata*, following the induction of superoxide dismutase production by the cells exposed to four different antibiotics (Aderemi et al., 2018). For some microalgae, pre-acclimation to sub-toxic concentrations of the EC may be required to initiate enzyme production in order to screen for biodegradation potential (Sutherland and Ralph, 2019). For example, microalgal biodegradation of several different antibiotics was enhanced following pre-exposure of the microalgal strain to low levels of the antibiotic due to increased production of antioxidants, including xanthophylls, by the cells (Chen et al., 2015; Xiong et al., 2017). Biodegradation may also lead to intermediary products that could be similarly, or more toxic, than the parent compound. Identification of the breakdown products with specific assays, coupled with toxicological screening is an important step that needs to be included in microalgal biodegradation assessments.

For microalgal species with demonstrated biodegradation capability, the induction of elevated Phase I and Phase II enzyme production can further enhance the EC degradation process, both improving its efficiency and effectiveness. This can be induced through genetic means, such as synthetic biology, targeted gene editing, or genetic engineering. For example, Zhang et al. (2018) used random mutagenesis and site-directed mutagenesis to increase the production of the degrading enzyme, laccase, by 31- to 37-fold in the white-rot fungus *Cerrena unicolor* BBP6. Similar approaches could be used to increase the biocatalytic activities of microalgal laccases.

Synthetic biology approaches (section “Synthetic Biology,” Figure 1a), can be used to engineer microalgae and overexpress entire artificial degrading pathways that include enzymes, such as fungal laccases, peroxidases, cellulases, and ligninases, to further increase the potential of algal bioremediation. These pathways can be either expressed in the host in the same configuration as in the source organism, or even in new-to-nature combinations, picking enzymes from multiple organisms and assembling new degradation pathways, both by rational design, and by random/combinatorial assembly and screening (Tay et al., 2017). While there are currently limited studies on genetic engineering of microalgae for bioremediation purposes, Chiaiese et al. (2011) successfully demonstrated fungal laccase POX A1b expression in the green alga, *Chlorella emersonii*, which enhanced microalgal biodegradation of phenols by up to about 40%. However, while genetically engineering microalgae for enhanced biodegradation appears promising, the potential environmental risks intrinsic to the use of genetically modified organisms (GMO) that would limits their application in outdoors settings need to be evaluated (Szyjka et al., 2017). In addition to this, for many countries, the legislation around the limited use, or the total ban of, GMOs means that transgenic microalgae for ECs biodegradation would not be a viable option, at present.

While microalgae have the demonstrated ability to biodegrade ECs associated with agricultural practises, further research is needed to exploit microalgal biodegradation, through enhanced enzyme expression and optimized growth conditions. When coupled with nutrient removal, such as HRAPs, microalgal treatment of EC can be a cost-effective viable option for the reduction of contaminant pollution in waterways (Sutherland and Ralph, 2019).

## Conclusion

Agriculture is one of the most ancient human practices and it has always been essential to our civilization. Agriculture and human society have co-evolved, reciprocally influencing each other. Over millennia humans isolated, bred, and generated new species to satisfy needs that have been steadily increasing in size and diversity. In modern times, agriculture technology has seen impressive improvements in yield, efficiency, and product differentiation thanks to developments in cultivation technology, genetics, and phenomics. Although algae-derived applications have been present in human history, the push to develop these organisms as industrial resources is a very recent objective. Compared to conventional agriculture crops, algae-based practices are an extremely young application field, and all current industrial algal strains are relatively uncharacterized. However, decades of foundational research on algal biochemistry and physiology (not reviewed here), may be leveraged to expedite the use of algae in biotechnology (Hildebrand et al., 2013). Efforts to progress the understanding of diverse algal traits has recently been bolstered by the advent of genome sequencing projects and functional genetic tools, revealing novel aspects of algal metabolism relevant to industrial applications (Moellering and Benning, 2010; Allen et al., 2011; Fabris et al., 2012; Kirst et al., 2012; Radakovits et al., 2012; Fabris et al., 2014; Abbriano et al., 2018; Luo et al., 2018; Pollier et al., 2019; Smith et al., 2019). Moreover, it is expected that knowledge on algal traits will be increasingly generated by the implementation of advanced synthetic and molecular biology approaches combined with phenomics. Presently, however, the relatively few algal species employed in commercial applications largely consist of natural isolates with minimal selections, breeding or genetic engineering (if any) to better perform in industrial settings or for improved yields. Despite this, as illustrated by the achievements highlighted in this review, algae already find applications in many industrial fields and sectors, often with the clear potential of replacing more energy, cost, and environmentally intensive solutions. Evaluating the current progress and achievement of algal biotechnology and industry from this perspective is at the same time both impressive and encouraging, and this needs to be kept into account when drawing the trajectory of future developments of this field. The emerging technologies that we described will drastically accelerate the process of industrialization of algae, providing knowledge and tools to deliver highly productive, algae-based solutions to a diversity of societal needs. This includes deeper understanding of algal biology, genetics, and biochemical capabilities, which will drive the optimization of both the organisms and the environment in which it is cultivated. This will allow in the near future the move toward *ad hoc*, highly productive strains, either as novel natural isolates or genetically engineered strains, and efficient cultivation systems with minimal environmental impact. We envision that high-tech algae-based solutions will find applications in almost every industrial sector, including ones essential to meeting the increasing needs of human society, such as food, pharmaceutical and bulk chemicals manufacture, while ensuring minimal environmental impact and lower production costs. The development of highly efficient algal biorefineries (’T Lam et al., 2018; Figure 2) will allow co-sourcing different products, minimizing waste and maximizing the productivity, improving the economics of processes otherwise low-efficient. As such, we anticipate that the progress of algae biotechnology will have a disruptive effect to the current industrial landscape, and will prompt the emergence of a scalable, sustainable, and efficient algae-based bio-economy, which will be key in overcoming challenges and limitations that conventional agriculture will face in the years ahead.

## Author Contributions

MF, RA, AC, DS, MP, LL, JM, UK, PaR, CH, and TK wrote the manuscript. All authors read and edited the manuscript.

## Conflict of Interest

The authors declare that the research was conducted in the absence of any commercial or financial relationships that could be construed as a potential conflict of interest.

## Footnotes

1 www.indexmundi.com
